# Intelligent detection and grading diagnosis of fresh rib fractures based on deep learning

**DOI:** 10.1186/s12880-025-01641-0

**Published:** 2025-03-24

**Authors:** Tongxin Li, Mingyi Liao, Yong Fu, Fanghong Zhang, Luya Shen, Junliang Che, Shulei Wu, Jie Liu, Wei Wu, Ping He, Qingyuan Xu, Yi Wu

**Affiliations:** 1 Department of Digital Medicine, College of Biomedical Engineering and Medical Imaging, Army Medical University, Third Military Medical University, Chongqing, China; 2 National Center for Applied Mathematics in Chongqing, Chongqing Normal University, Chongqing, China; 3 Department of Cardiothoracic Surgery, Dianjiang People’s Hospital of Chongqing, Chongqing, China; 4 Department of Thoracic Surgery, Southwest Hospital, Army Medical University, Third Military Medical University, Chongqing, China; 5 Department of Cardiac Surgery, Southwest Hospital, Army Medical University, Third Military Medical University, Chongqing, China

**Keywords:** Artificial intelligence, Deep learning, Fresh rib fracture, Detection, Grading diagnosis

## Abstract

**Background:**

Accurate detection and grading of fresh rib fractures are crucial for patient management but remain challenging due to the complexity of rib structures on CT images.

**Methods:**

Chest CT images from 383 patients with rib fractures were retrospectively analyzed. The dataset was divided into a training set (*n* = 306) and an internal testing set (*n* = 77). An external testing set of 50 patients from the public RibFrac dataset was included. Fractures were classified into severe and non-severe categories. A modified YOLO-based deep learning model was developed for detection and grading. Performance was compared with thoracic surgeons using precision, recall, mAP50, and F1 score.

**Results:**

The deep learning model showed excellent performance in diagnosing fresh rib fractures. For all fractures types in internal test set, the precision, recall, mAP50, and F1 score were 0.963, 0.934, 0.972, and 0.948, respectively. The model outperformed thoracic surgeons of varying experience levels (all *p* < 0.01).

**Conclusion:**

The proposed deep learning model can automatically detect and grade fresh rib fractures with accuracy comparable to that of physicians. This model helps improve diagnostic accuracy, reduce physician workload, save medical resources, and strengthen health care in resource-limited areas.

**Clinical trial number:**

Not applicable.

**Supplementary Information:**

The online version contains supplementary material available at 10.1186/s12880-025-01641-0.

## Introduction

Rib fractures are the most common injuries in patients with blunt chest trauma [1, 2] and may be accompanied by severe complications such as pulmonary contusion, pneumonia, and hemopneumothorax [3, 4]. The number and displacement of rib fractures, as well as the presence of associated organ injuries, are correlated with mortality [5, 6]. Therefore, accurately identifying the number and severity of rib fractures is crucial.

With the increased use of chest multidetector computed tomography scans, the detection rate of rib fractures has significantly improved [7]. Compared to X-ray, CT offers greater contrast and resolution, providing a comprehensive view of the fracture site and enabling the detection of minor lesions and the assessment of other complications. However, the diversity and complexity of rib fracture shapes make it challenging to identify all fractures across hundreds of thin-slice CT images, often leading to missed diagnoses. The literature reports that 20.7% of initial chest CT scan reports misidentify the number or location of fractured ribs [8], and even after rib reconstruction, 20.9% of rib fractures are missed [9].

Recently, deep learning algorithms have been applied in the field of medical image processing [10], including image registration [11, 12], detection [13–15], segmentation [16–18] and disease prognosis [19, 20]. Notably, studies focusing on Alzheimer’s disease [21] and brain tumor classification [22], have highlighted the potential of next-generation convolutional architectures to enhance diagnostic accuracy and efficiency. Building upon these advancements, our study applies a deep learning approach to the detection and grading of fresh rib fractures. This approach not only aims to assist clinicians in identifying fractures more accurately but also seeks to alleviate their workload and ultimately improve patient outcomes. Several studies have reported promising results in rib fractures detection [23–26]. Azuma et al. [27] developed and validated a convolutional neural network (CNN) model for detecting rib fractures, showing that the CNN model could enhance the diagnostic ability of radiologists for any type of rib fracture. Zhou et al. [26] included clinical information in their CNN model, further improving diagnostic efficiency and reducing diagnosis time. However, the importance of fractures varies with their type. Chronic rib fractures usually do not require clinical intervention and do not affect treatment decisions or patient prognosis. In contrast, fresh rib fractures often exhibit severe displacement and may be accompanied by organ injuries, especially severely displaced and comminuted rib fractures, which are often accompanied by fatal complications such as severe pulmonary infection, persistent hemothorax, and massive hemoptysis [28]. Moreover, rapid and accurate detection of the severity of fresh rib fractures helps to implement necessary treatment measures, thereby improving patient prognosis. Therefore, accurately identifying the location and severity of fresh rib fractures is particularly important. To the best of our knowledge, there is a scarcity of studies focusing on the graded diagnosis of fresh rib fractures, with an emphasis on those that are severely displaced.

In our study, we developed a deep learning-based model for the intelligent detection of fresh rib fractures and graded diagnoses based on the severity of rib displacement, categorizing fractures into severe and non-severe. We conducted a comprehensive analysis of different subgroups of fracture severity and compared the model’s performance with that of experienced thoracic surgeons.

## Methods

### Data sets and classification criteria

A retrospective collection of initial chest CT scans of patients diagnosed with rib fractures from January 2021 to April 2023 was conducted at the First Affiliated Hospital of Army Medical University (Hospital A) and Chongqing Dianjiang People’s Hospital (Hospital B). This study obtained ethical approval from the Ethics Committee of the First Affiliated Hospital of Army Medical University (ID: KY2023062) and Chongqing Dianjiang People’s Hospital (ID: DYLL-LW-2023-03). All patient data were de-identified prior to analysis, and this retrospective study was conducted in accordance with the Declaration of Helsinki. The inclusion criteria were patients aged ≥ 18 years with a history of trauma. Exclusion criteria included: (1) old or healed fractures, (2) artifacts, (3) bone tumors or bone destruction, (4) postoperative patients.

**Fig. 1:**
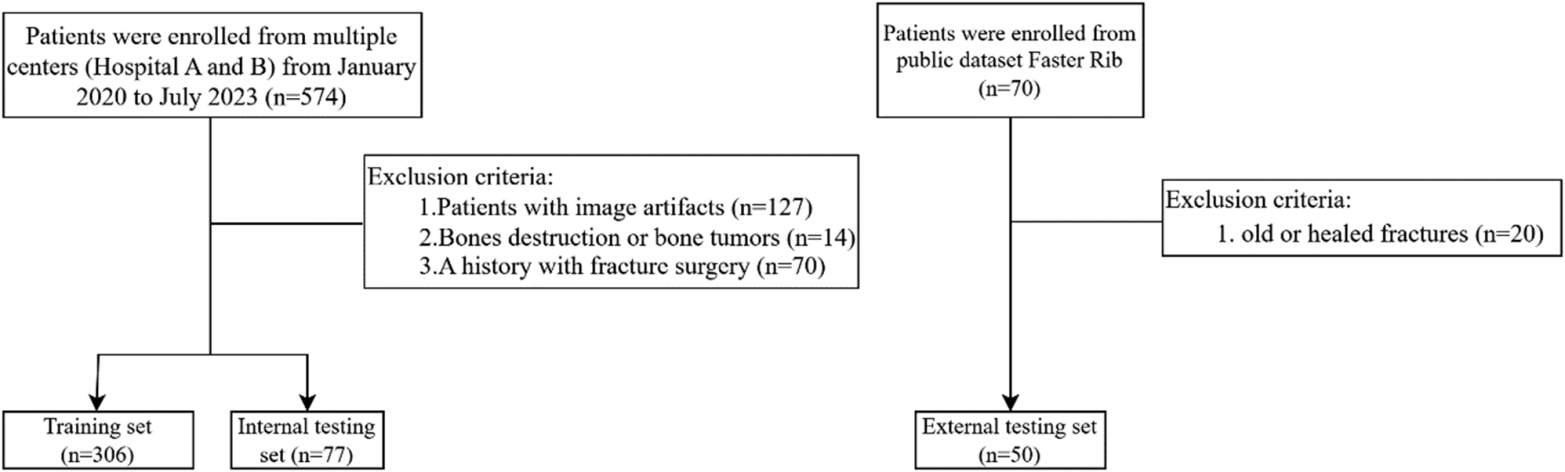
Flow chart of patient selection

A total of 383 patients from the two centers were randomly divided into a training set (*n* = 306) and an internal testing set (*n* = 77). A rigorous randomization method was employed to minimize bias introduced by data partitioning and ensure the reliability and scientific validity of the study results. The external testing dataset comprised 50 CT scans from the public RibFrac dataset [29], and radiologists re-annotated the CT scans of these 50 cases. The specific screening criteria are shown in Fig. 1.

**Fig. 2:**
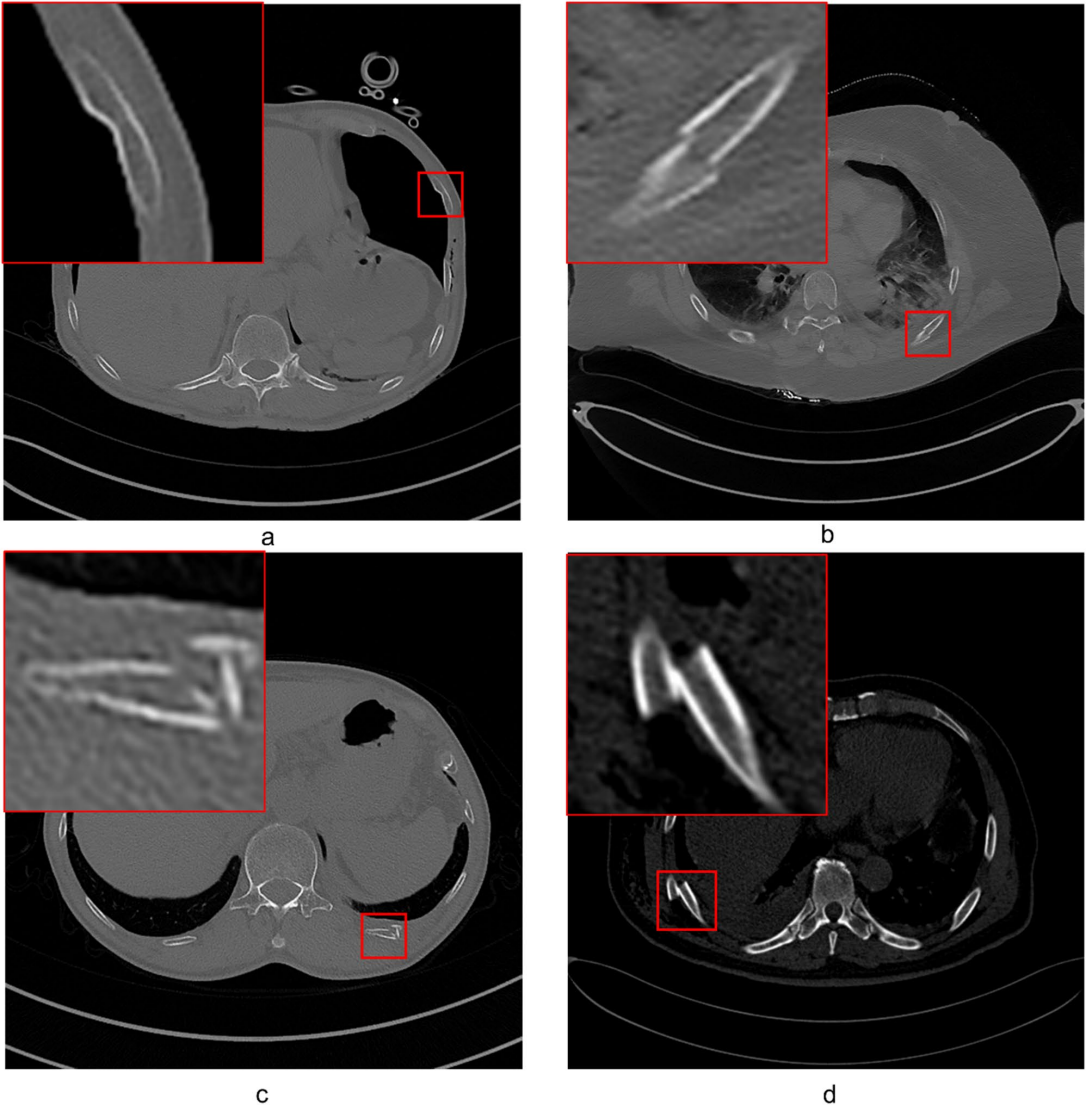
Examples of two different types of rib fractures. The top row shows non-severe fractures, while the bottom row shows severe fractures. (**a**) Mild fracture with cortical distortion, where the outer layer of the rib is slightly disrupted but remains mostly intact. (**b**) Mild fracture with misalignment or displacement, but the displacement distance is less than the rib’s diameter. (**c**) Severe comminuted fracture, with the rib broken into multiple fragments. (**d**) Severe fracture with significant displacement, where the displacement distance exceeds the rib’s diameter

In this study, fractures were classified into non-severe and severe based on the severity. Non-severe fractures were defined as: (1) fissure fractures with bone defects between the ends of the cortical bone without displacement, (2) cortical bone distortion or density changes, (3) fractures with angulation or displacement less than the rib diameter. Severe fractures were defined as: (1) fractures with displacement greater than the rib diameter, (2) fractures with observed bone fragments or comminuted fractures (Fig. 2).

### CT acquisition

Patients were positioned supine, with their arms raised above their heads (for those unable to do so due to shoulder or upper limb injuries, arms were placed naturally at their sides). The scanning range extended from the thoracic inlet to the lung bases or the entire ribcage. The scan was performed during a single breath-hold following inhalation. Two different CT scanners were utilized for the examinations: Philips Brilliance 16 (Philips Medical Systems) and SOMATOM Definition (Siemens Healthineers). The tube voltage was set to 120 kV, with automatic modulation of the tube current. The slice interval ranged from 1 to 5 mm, and the slice thickness varied between 1 and 5 mm.

### Image annotation

The CT slices in the training set were independently annotated by a thoracic surgeon with 5 years of experience and a radiologist with 5 years of experience. These two physicians used Makesense (https://www.makesense.ai/) to mark rib fractures with rectangular boxes and used different colors to indicate severity (Supplementary Fig. 1). In cases of uncertainty regarding fracture type, the two physicians discussed until a consensus was reached, or a third physician was involved to reach a final agreement. Annotations were made without access to any clinical information of the patients, and these annotations were used as the ground truth for training the automated detection algorithm.

The internal and external test datasets were annotated independently by another three thoracic surgeons -a junior, a middle grade, and a senior, and these annotations were subsequently compared with the model’s predictions.

### Model architecture

The model architecture: You-Only-Look-Once (YOLO) version 8 [30] is a real-time object detection algorithm that can detect and locate multiple objects in images or videos with relatively fast speed. While maintaining high detection accuracy, it significantly improves reasoning speed and is suitable for various computing devices, including mobile devices and embedded systems. The model architecture consists of three main components: the Backbone, the Neck, and the Head networks.

Although YOLOv8 has achieved notable success in fields such as autonomous driving and industrial inspection, it still exhibits significant shortcomings in the detection of small targets in medical imaging. To enhance YOLOv8’s capability in detecting small targets, we propose improvements by incorporating high-resolution feature maps and introducing a more sophisticated Feature Pyramid Network (FPN). The specific innovations are as follows:

1. Design of the C2f_EMA Module in YOLOv8: A C2f_EMA module was designed in YOLOv8, which incorporates the EMA (Efficient Multi-scale Attention) attention mechanism. This modular design retains the original feature extraction capability of the C2f module while enhancing feature representation through the attention mechanism.

2. Integration of the Attention Mechanism: By embedding the EMA attention mechanism into the C2f module of YOLOv8, the study achieves dynamic adjustment of channel and spatial dimension weights during the feature extraction and fusion stages, thereby improving the model’s discriminative ability.

3. Improved Feature Pyramid Network: The study enhances the FPN-PAN structure of YOLOv8 by introducing the new C2f_EMA module. This improvement facilitates the construction of a more efficient feature pyramid network, leading to enhanced model performance.

**Fig. 3:**
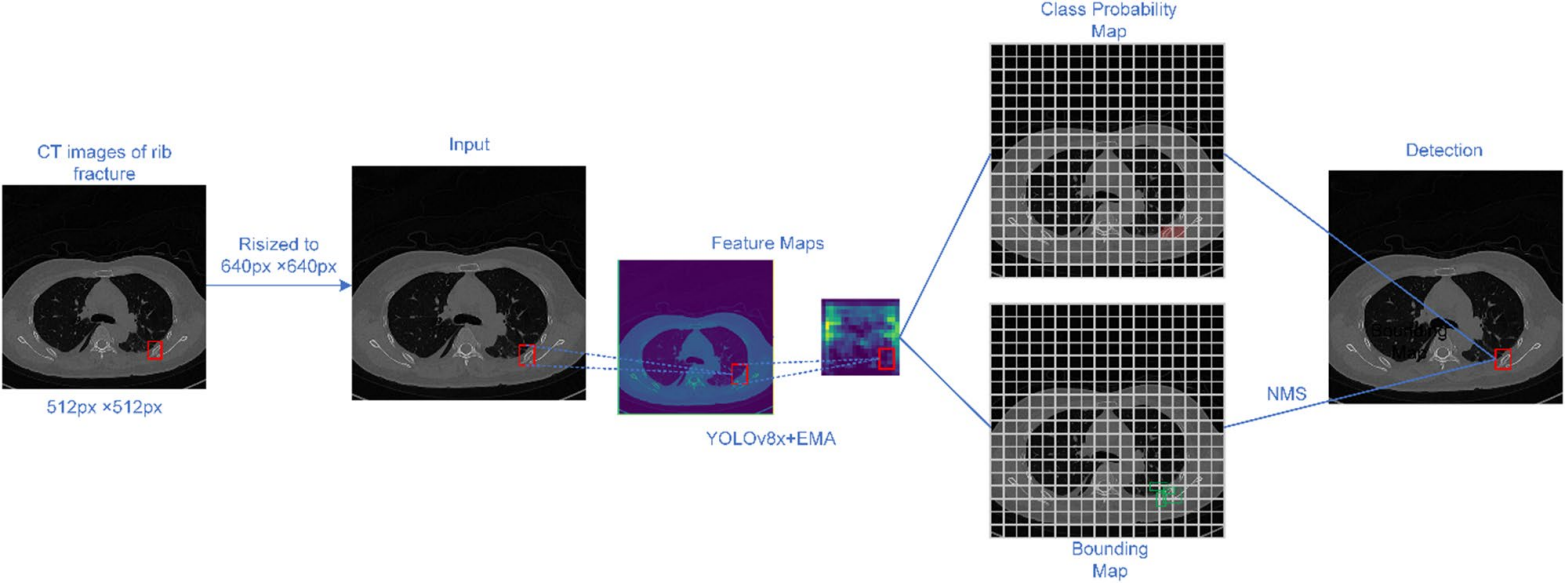
Rib fracture detection in CT images using a YOLO-based model with Exponential Moving Average (EMA). The input CT images are resized to 640px × 640px and processed through the model to generate feature maps. These maps are used to produce a class probability map and a bounding map, which are then passed through Non-Maximum Suppression (NMS) to yield the final detection of the fracture location

Figure 3 illustrates the process of detecting rib fractures using the improved YOLOv8 model architecture with the C2f_EMA module and enhanced Feature Pyramid Network, showcasing how feature maps are processed and the final detection result is achieved. These innovations collectively aim to address the limitations of YOLOv8 in small target detection, particularly in the context of medical imaging, by improving feature extraction, fusion, and multi-scale representation capabilities. These innovations collectively aim to address the limitations of YOLOv8 in small target detection, particularly in the context of medical imaging, by improving feature extraction, fusion, and multi-scale representation capabilities.

**Fig. 4:**
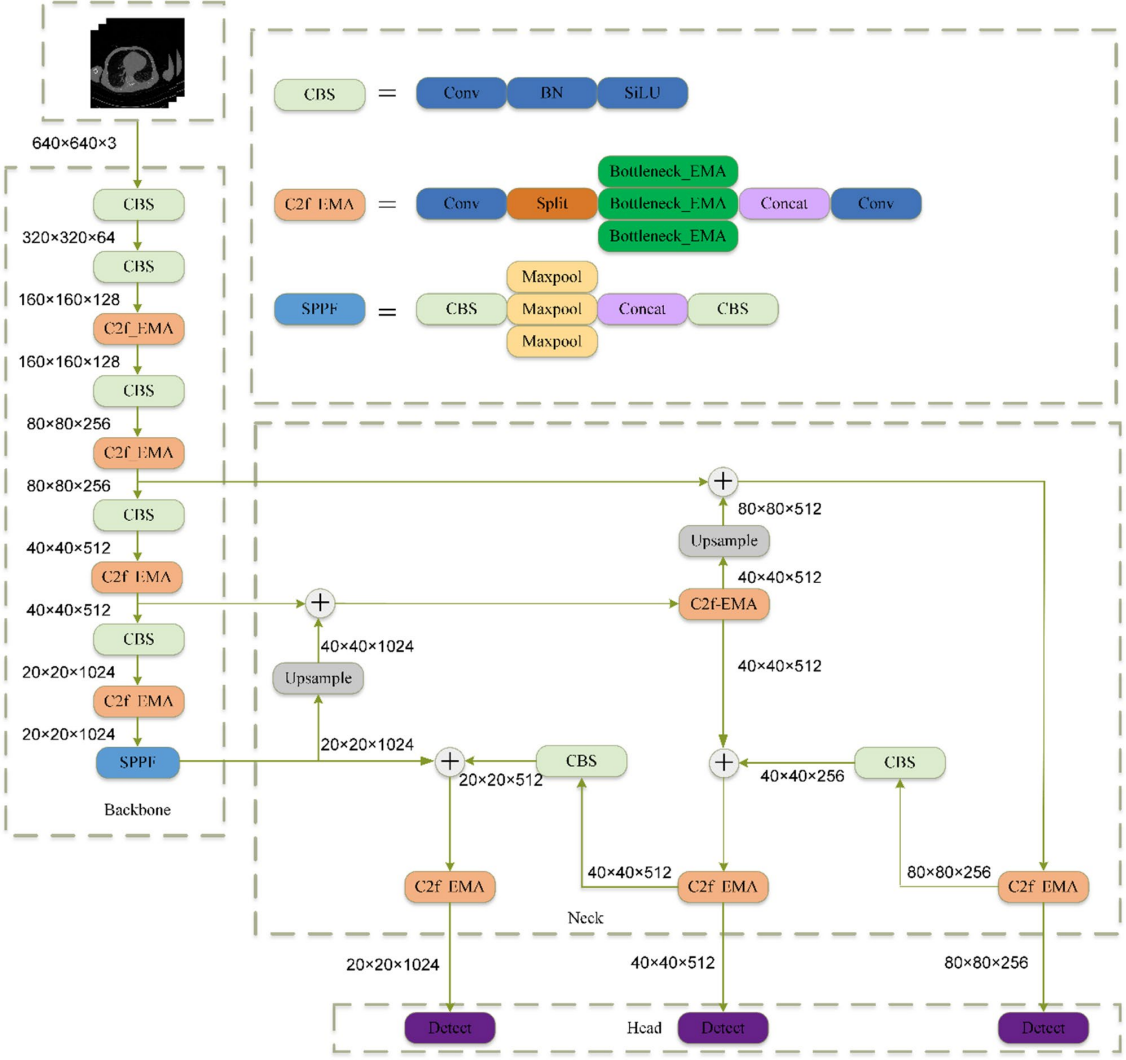
The architecture of a YOLO-based model incorporating Exponential Moving Average (EMA). The backbone extracts features using CBS and C2f_EMA modules, followed by spatial pyramid pooling (SPPf) and feature fusion in the neck. The detection head generates outputs across multiple scales for object detection

As shown in Fig. 4, the input image is first passed through the Backbone to extract multi-scale features. The Backbone network (e.g., CSPDarknet53) generates multi-scale feature maps through convolutional layers and downsampling operations [31, 32] capturing semantic information from low-level to high-level layers, thereby enhancing the feature extraction capability for small targets. To improve feature representation, we introduce the C2f_EMA module, which combines the C2f structure with the EMA (Efficient Multi-scale Attention) mechanism to dynamically adjust channel and spatial weights, enhancing the focus on important features.

Subsequently, the multi-scale features are fed into the Neck module for feature fusion. The Neck adopts the FPN (Feature Pyramid Network) and PAN (Path Aggregation Network) structures, aggregating features through top-down and bottom-up paths to enhance multi-scale object detection capabilities. We also integrate the C2f_EMA module into the Neck, further optimizing the feature pyramid network and improving feature fusion performance.

Through the collaborative work of the Backbone and Neck, combined with the C2f_EMA module, the model can more efficiently extract and fuse multi-scale features, providing richer feature representations for the detection head (Head). This significantly improves the accuracy and robustness of small target detection, particularly excelling in complex scenarios such as medical imaging.

$$\:{F}_{b}=Backbone\left(x\right)$$

$$\:{F}_{n}=FPN-PAN\left({F}_{b}\right)$$

$$\:Y=Head\left({F}_{n}\right)$$

$\:x\in\:{\mathbb{R}}^{H\times\:W\times\:3}$, where Fb represents multi-scale features, Fn is the fused feature map, and Y is the output of the detection head.

### Model evaluation

To objectively assess the model’s performance, four evaluation metrics were calculated: mean average precision (mAP), precision, recall, and F1 score. For localization accuracy, mAP was used, which is a standard metric in artificial intelligence. The areas under the precision-recall curve (AUPRC), with values ranging between 0 and 1. To calculate mAP, intersection of union (IoU) is used, measuring the overlap between predictions and ground truth. We defined the IoU threshold as 0.5 to classify the prediction box as a true positive or false positive. An IoU of 0.5 was chosen because, in clinical CT examinations, such a prediction box can already prompt clinicians (Supplementary Fig. 2). Precision is defined as the number of correctly predicted slices divided by the total number of predicted slices, while recall is defined as the number of samples correctly predicted as positive by the model divided by the total number of positive samples. The F1 score is the harmonic mean of precision and recall, providing a balance between the two metrics. It is particularly useful when the class distribution is imbalanced.

### Statistical analysis

This study employed SPSS (version 26.0, IBM, NY for statistical analysis, with a two-tailed p-value threshold of < 0.05. Normality of continuous variables was assessed using the Kolmogorov-Smirnov test. Normally distributed data were presented as mean ± standard deviation, otherwise, M (P25-P75) were utilized. Categorical variables were expressed as frequencies and percentages.

For normally distributed data, two-sample t-tests were applied, and non-normally distributed data were analyzed using the Mann-Whitney U test. Categorical variables were compared using Chi-squared or Fisher’s exact test. The z-test was used to compare whether there was a statistical difference between the system and the thoracic surgeons.

## Results

### Patient characteristics

This study included data from 383 patients with rib fractures from two centers, comprising a total of 4673 annotations. Among these annotations, 3,043 were for non-severe fractures and 1,630 for severe fractures. Of the 383 patients, 130 were from Hospital A and 253 from Hospital B. We randomly selected 306 patients for the training set and 77 patients for the internal testing set. Our results showed no statistically significant differences in gender (*P* = 0.256) and age (*P* = 0.766) between the training and testing sets (Table 1). Additionally, there were no significant differences in age (*P* = 0.565) between patients from the two centers, and hospital A had a significantly higher proportion of male patients compared to hospital B (*P* < 0.01) as detailed in Supplementary Table 1.

**Table 1:** Patient’s radiologic and clinical information in training, internal and external testing datasets

Characteristic	Training data set	Internal testing data set	External testing data set	*P* value
No. of patients, n (%)	306	77	50	-
Age, years, M (Q1, Q3)	57 (48 ~ 69)	55 (53 ~ 62)	-	0.256
Gender, male, n (%)	202 (66.0%)	59 (76.6%)	-	0.766
No. of fracture annotations (total)	3646	1027	821	-
Annotations of non-severe fracture	2455	685	660	-
Annotations of Severe fracture	1191	342	161	-

The training set consisted of 306 patients, with a total of 3,646 annotations, including 2,455 non-severe fractures and 1,191 severe fractures. The internal testing set included 77 patients, with a total of 1,027 annotations, comprising 685 non-severe and 342 severe fractures. Additionally, we incorporated 50 patients from the public RibFrac dataset, with a total of 821 annotations, including 660 non-severe fractures and 161 severe fractures.

### Performance of the models

In the internal test set, the overall precision for all fracture types was 0.963, recall was 0.934, mAP50 was 0.972, and the F1 score was 0.948. For external test dataset, precision was 0.880, recall was 0.796, mAP50 was 0.857, and F1 score was 0.836 (Table 2).

**Table 2:** Model performance internal and external data sets

Fracture type	Precision	Recall	mAP50	F1 score
Internal Testing data set	0.963	0.934	0.972	0.948
Non-Severe Fracture	0.957	0.909	0.964	0.932
Severe Fracture	0.968	0.959	0.981	0.963
External Testing data set	0.880	0.796	0.857	0.836
Non-Severe Fracture	0.867	0.774	0.843	0.818
Severe Fracture	0.892	0.819	0.872	0.854

**Fig. 5:**
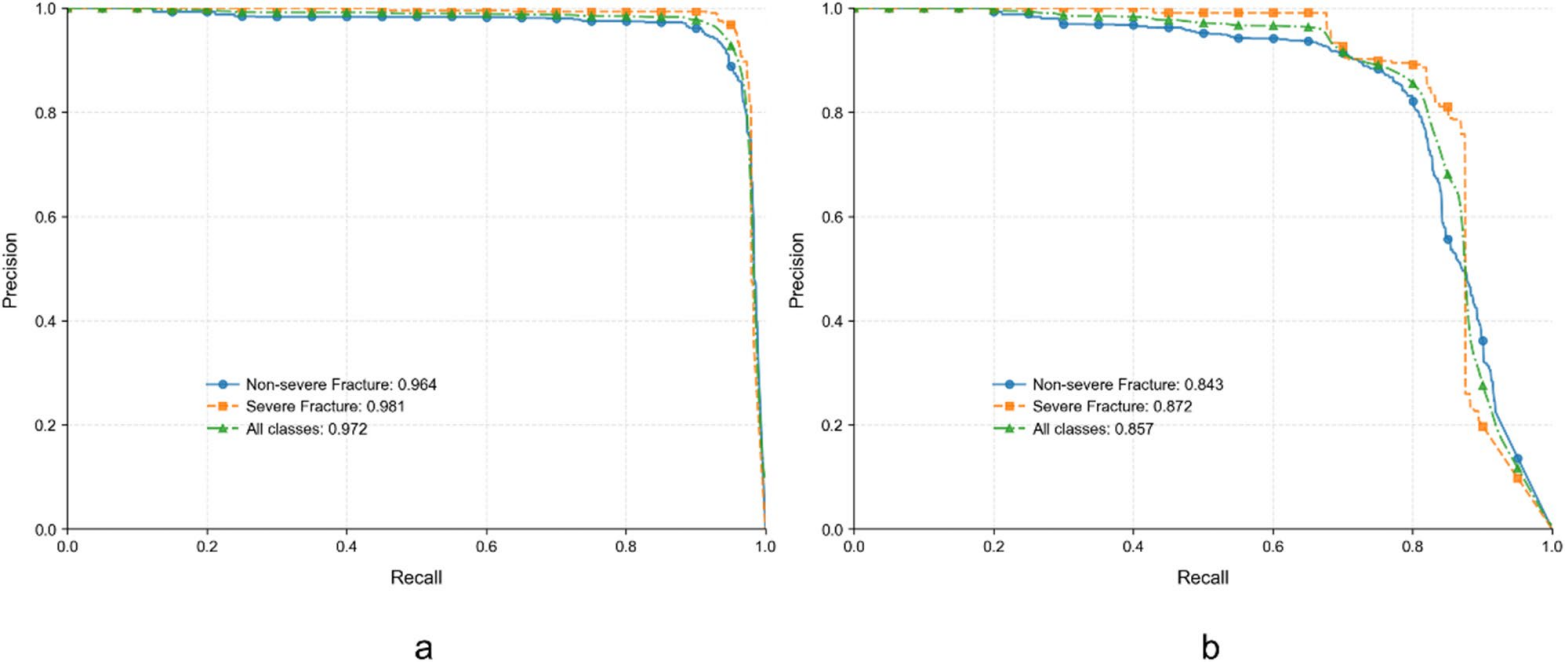
Precision-Recall curves depicting the model’s diagnostic performance for fracture identification. (**a**) Internal test set results: The model demonstrates high Average Precision (AP) scores, with an AP of 0.964 for non-severe fractures and 0.981 for severe fractures, culminating in a combined AP of 0.972 for all classes. (**b**) External test set results: The model maintains robust performance with APs of 0.843 for non-severe fractures, 0.872 for severe fractures, and an aggregate AP of 0.857 across all classes

**Fig. 6:**
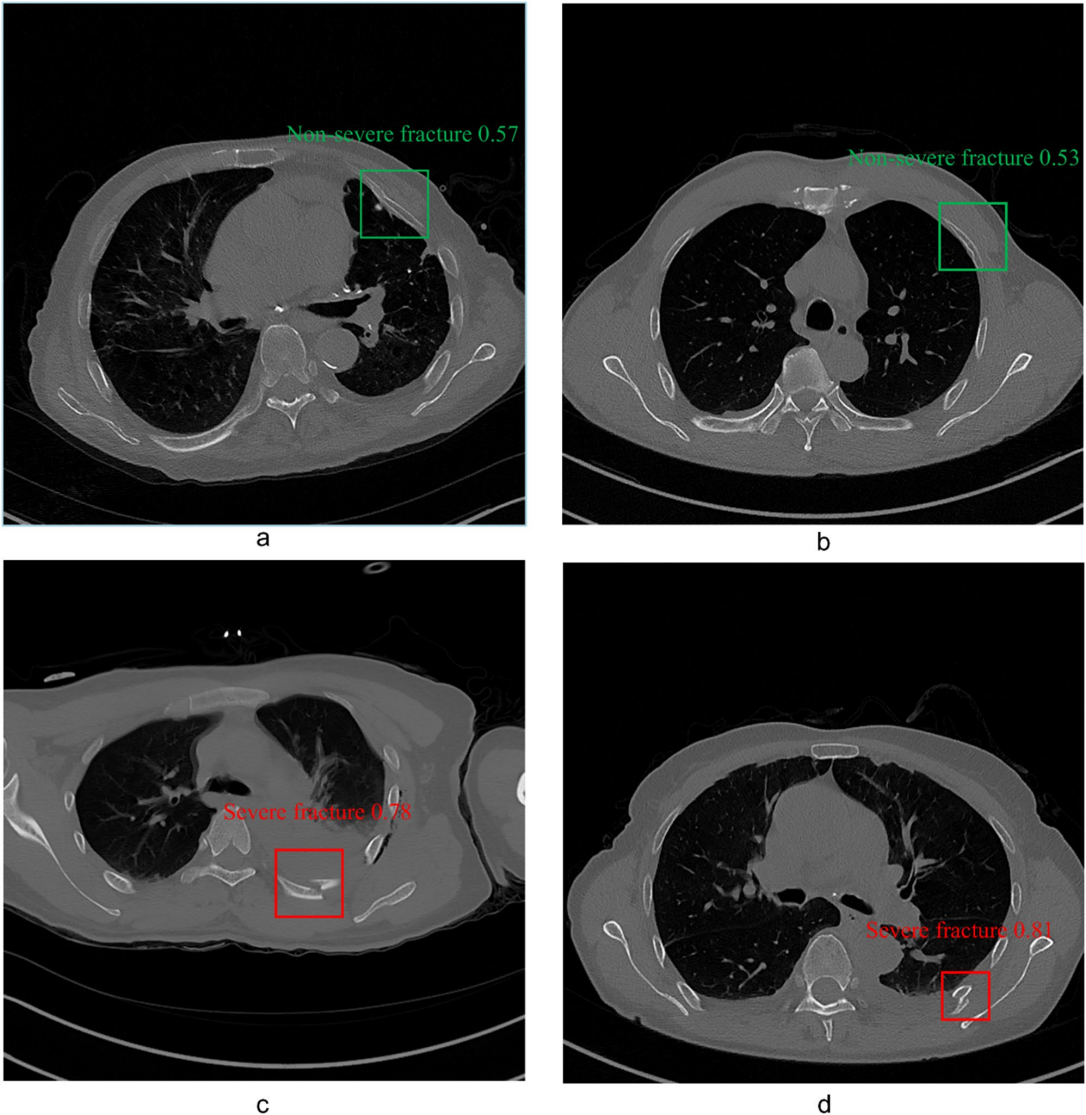
Overview of fracture cases identified by the model, encompassing non-severe and severe fracture recognition cases. **a** and **b**: Non-severe fractures identified by the model, Fig. 6c and d: Severe fractures identified by the model

We visualized the model’s performance using the precision-recall (PR) curve. Figure 5a illustrates the PR curves for the internal test set, where the AUPRC values are 0.964 for non-severe fractures, 0.981 for severe fractures, and 0.972 for all classes combined. Figure 5b shows the PR curves for the external test set, with AUPRC values of 0.843 for Non-severe fractures, 0.872 for Severe fractures, and 0.857 for all classes combined. These results demonstrate that our model exhibits excellent performance on the external test set, indicating good generalizability. Figure 6 presents examples of rib fractures successfully identified by our model. Additionally, a confusion matrix has been created to illustrate the model’s performance on the internal and external testing set (Supplementary Fig. 3).

To further demonstrate the effectiveness of the model, we performed comparative experiments with the YOLOv8x and EfficientVit-M3 models, respectively, for the two different datasets and for non-severe fractures and severe fractures. The experimental results show that the F1-score of the proposed model on the internal verification set is higher than other models, and the accuracy and recall rate of the proposed model on the external verification set are 5% higher than other models, which further indicates the effectiveness of the model in detecting small targets (Table 3).

**Table 3:** Performance comparison of rib fracture detection models on internal and external testing datasets

Model		Internal testing data set				External testing data set			
		Precision	Recall	mAP50	F1 score	Precision	Recall	mAP50	F1 score
yolov8x-DAttention	All classes	0.869	0.829	0.897	0.848	0.794	0.657	0.758	0.718
	Non-severe fracture	0.845	0.829	0.892	0.838	0.742	0.667	0.735	0.700
	Severe fracture	0.893	0.828	0.901	0.860	0.846	0.646	0.780	0.732
efficientVit-M3	All classes	0.894	0.782	0.882	0.834	0.642	0.485	0.517	0.554
	Non-severe fracture	0.860	0.762	0.857	0.806	0.580	0.411	0.450	0.480
	Severe fracture	0.929	0.801	0.908	0.860	0.703	0.559	0.584	0.620
YOLOv8x	All classes	0.828	0.826	0.889	0.824	0.777	0.618	0.731	0.688
	Non-severe fracture	0.766	0.816	0.865	0.788	0.725	0.596	0.685	0.654
	Severe fracture	0.890	0.836	0.914	0.862	0.829	0.64	0.777	0.722
Ours	All classes	0.963	0.934	0.972	0.948	0.880	0.796	0.857	0.836
	Non-severe fracture	0.957	0.909	0.964	0.932	0.867	0.774	0.843	0.818
	Severe fracture	0.968	0.959	0.981	0.963	0.892	0.819	0.872	0.854

### Comparison of the performance between our model and thoracic surgeons

**Fig. 7:**
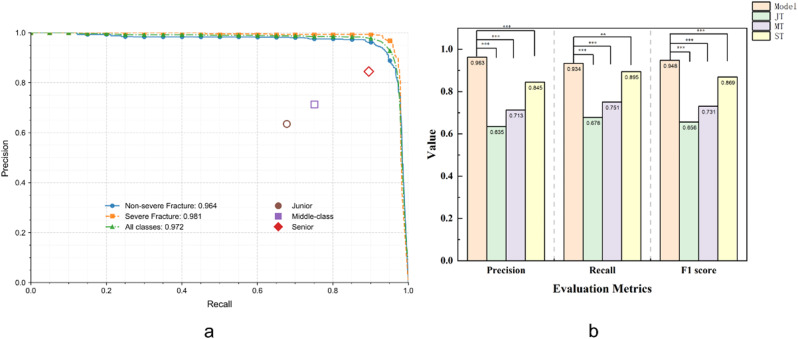
Performance comparison between our model and doctors. (**a**): doctor’s performance on the precision-recall curve. (**b**): The Statistical results of precision, recall and F1 score on internal test dataset. mAP: mean average precision, JT: Junior Thoracic surgeon, MT: Middle grade Thoracic surgeon, SR: Senior Thoracic surgeon, Model: Deep learning system for fresh rib fracture detection and grading model

Table 4, and Fig. 7 present a comparison of precision, recall, and F1 score between three thoracic surgeons of varying experience levels and our model. For non-severe fractures, the F1 score ranged from 0.660 to 0.862 across surgeons, with senior surgeon achieving the highest score. For severe fractures, senior surgeon again performed the best with an F1 score of 0.880, while junior surgeon had the lowest at 0.648. In overall performance, senior surgeon led with an F1 score of 0.869, while our model outperformed all surgeons in both precision and recall, demonstrating its superior capability in rib fracture detection. These results emphasize the model’s potential in improving detection accuracy compared to experienced clinicians.

**Table 4:** Comparison of diagnostic performance between thoracic surgeons and our model

	Senior Surgeon			Middle grade surgeon			Junior surgeon		
Fracture type	Precision	Recall	F1 score	Precision	Recall	F1 score	Precision	Recall	F1 score
Non-severe fracture	0.802	0.932	0.862	0.669	0.736	0.701	0.651	0.670	0.660
Severe fracture	0.922	0.842	0.880	0.793	0.774	0.783	0.606	0.695	0.645
All classes	0.845	0.895	0.869	0.713	0.751	0.731	0.635	0.678	0.656

## Discussion

Chest CT scans are often the preferred imaging modality for patients with chest trauma because they can identify many injuries that may be missed by chest X-rays, including pulmonary contusions, hemothorax, pneumothorax, and rib fractures. Moreover, rib fractures are considered indicators of severe trauma [33]. Traditional detection methods require physicians to meticulously evaluate the entire CT scan, which is a time-consuming and error-prone process, particularly for less experienced radiologists or thoracic surgeons. Additionally, the number and displacement of rib fractures are related to the follow-up treatment plan [34, 35]. Therefore, thoracic surgeons should prioritize different types of rib fractures. Chronic rib fractures, characterized by the presence of a mature callus or an invisible fracture line, do not require clinical intervention and do not affect treatment decisions or patient prognosis, making their detection unnecessary. In contrast, timely identification and localization of fresh rib fractures are crucial, especially in emergency settings, as severely displaced rib fractures may be associated with internal organ injuries, significantly impacting patient outcomes [36].

To address this issue, we developed a deep learning-based intelligent diagnostic model for the classification and detection of fresh rib fractures, which was validated on both internal multicenter datasets and external public datasets. The model outperformed experienced thoracic surgeons and demonstrated exceptional performance.

Recently, Yao et al. [37] developed a deep learning-based rib fracture detection system that achieved high-performance detection and diagnosis of rib fractures on chest CT images, significantly reducing physician workload and minimizing misdiagnoses. However, this study only addressed the binary classification of fracture presence. Zhou et al. [38] developed a convolutional neural network (CNN) model that classified fractures into fresh, healing, and old fractures but did not perform a graded diagnosis for precise fracture diagnosis. Zhou et al. [26] reported the use of clinical information in their CNN model, which similarly improved diagnostic efficiency and reduced diagnosis time. Xiong et al. [39] found that the performance of radiologists on night shifts was inferior to that on day shifts, and the use of a deep learning-based computer-aided diagnosis (CAD) system for rib fractures helped night shift radiologists achieve performance levels comparable to their daytime performance. Unlike previous studies, our research focused on fresh rib fractures, as their rapid localization and diagnosis are crucial components of intelligent diagnosis and treatment of acute chest trauma. Chronic rib fractures do not affect treatment decisions or patient outcomes, making the focus on fresh rib fractures more aligned with real clinical scenarios. Our model can also intelligently grade fractures based on their severity, aiding in treatment decisions and prognosis evaluation for posttraumatic rib fractures.

Our model’s performance is also comparable to that of physicians, making it suitable as the “first reader.” This approach can help improve diagnostic accuracy, reduce diagnosis time, and reduce the workload of physicians, additionally, it can aid in building medical resources in under resourced areas.

In our model improvements, we optimized the Backbone, Neck, and Head networks to enhance feature extraction, fusion, and detection capabilities. The Backbone, designed to extract deep features from medical images, alternates between CBS modules (Convolution, Batch Normalization, and SiLU activation) and C2f_EMA modules, which include convolution layers and parallel Bottleneck_EMA branches for richer feature representation. It concludes with the SPPF module, which combines multiple Maxpool and Concat operations to capture critical global information. In the Neck, we focused on refining and consolidating multi-scale features using an Upsample operation and the C2f_EMA module to merge and process features across different levels, improving detection accuracy and robustness. Finally, the Head network, composed of additional C2f_EMA modules, integrates and processes refined features before passing them to the Detect layer, where bounding boxes and class predictions are generated. This comprehensive multi-level integration allows our model to achieve high-precision detection in medical images.

### Limitations of the study

Our study had several limitations. First, while the model can mark the fracture locations on CT images, it cannot provide the specific anatomical details, such as identifying which rib is fractured. Second, our sample size could be further expanded, and we plan to include more centers in future studies. Finally, prospective studies remain relatively scarce. We aim to collect more data and conduct prospective studies to further validate and optimize the model.

## Conclusion

In summary, we developed a deep learning-based intelligent detection model for the detection and grading of fresh rib fractures. The model demonstrated high fracture detection rates and localization accuracy. It is suitable as a “first reader” to assist physicians in quickly and accurately determining the condition of patients with rib fractures, reducing diagnostic time, improving diagnostic accuracy, and enhancing physician efficiency. Additionally, the model can help strengthen medical resources in under resourced areas, including rural and township regions.

## Electronic supplementary material

Below is the link to the electronic supplementary material.

Supplementary Material 1

Supplementary Material 2

Supplementary Material 3

Supplementary Material 4

Supplementary Material 5

## Data Availability

The datasets used and analysed during the current study are available from the corresponding author on reasonable request.

## Abbreviations

AI: Artificial intelligence
CT: Computed tomography
CNN: Convolutional neural network
CAD: Computer-aided diagnosis
mAP: mean average precision
ROC-AUC: Receiver operating characteristic - area under curve
PR: Precision-recall
IoU: Intersection over Union
EMA: Exponential moving average
CBS: Convolution, batch normalization, SiLU activation
SPPF: Spatial pyramid pooling in feature
YOLO: You only look once
AUPRC: Area under the precision-recall curve
