# Supplementary material for: Intelligent detection and grading diagnosis of fresh rib fractures based on deep learning
Source: BMC Med Imaging. 2025 Mar 24;25:98. doi: 10.1186/s12880-025-01641-0 (PMC11934624; doi:10.1186/s12880-025-01641-0)
Supplement: Supplementary file 1 — Supplementary Material 1 [file 12880_2025_1641_MOESM1_ESM.docx]

Legend of Supplementary Material

Supplementary Figure 1. Using Makesense to Annotate the Process of Rib Fracture. This image shows the annotation process of rib fractures using Makesense. The image on the right displays a CT scan of a rib fracture, with two annotations visible. The yellow box represents a non-severe fracture (label 0), and the red box represents a severe fracture (label 1). The left panel displays the image thumbnails, and the right panel shows the selected annotation tools, including options for points, lines, and polygons. This annotation process helps in marking and categorizing fractures based on severity, which is crucial for training deep learning models for automated detection.

Supplementary Figure 2. Rib fracture computed tomography (CT) images with varying intersections of union (IoUs). Red box is expert annotation and green box is predicted by artificial intelligence (AI). Figure 4A: IoU=0.3; Figure 4B: IoU=0.5; Figure 4C: IoU=0.8. In this study, we set the IoU threshold as ≥ 0.5 for true positive on object detection.

Supplementary Figure 3. An overview of Confusion Matrix. (a): Internal test data; (b): External test data.
