# Supplementary material for: Intelligent detection and grading diagnosis of fresh rib fractures based on deep learning
Source: BMC Med Imaging. 2025 Mar 24;25:98. doi: 10.1186/s12880-025-01641-0 (PMC11934624; doi:10.1186/s12880-025-01641-0)
Supplement: Supplementary file 2 — Supplementary Material 2 [file 12880_2025_1641_MOESM2_ESM.docx]

Supplementary Table 1. Patient’s radiologic and clinical information in two hospitals.

| Characteristic | Hospital A | Hospital B | P value |
| --- | --- | --- | --- |
| No. of patients, n (%) | 140 | 243 |  |
| Age, years, M (Q1, Q3) | 57 (50~67) | 56 (49~68) | 0.565 |
| Gender, male, n (%) | 113 (80.7%) | 153 (63.0%) | <0.01 |
| No. of fracture annotations (total) | 1503 | 3170 |  |
| Annotations of non-severe fracture | 971 | 2072 |  |
| Annotations of severe fracture | 532 | 1098 |  |

Hospital A: Southwest hospital; hospital B: Dianjiang People's Hospital of Chongqing; No: Number; M (Q1, Q3): Median (First Quartile, Third Quartile); CT: Computed Tomography.
