# Supplementary figures and images for: Intelligent detection and grading diagnosis of fresh rib fractures based on deep learning
Source: BMC Med Imaging. 2025 Mar 24;25:98. doi: 10.1186/s12880-025-01641-0 (PMC11934624; doi:10.1186/s12880-025-01641-0)

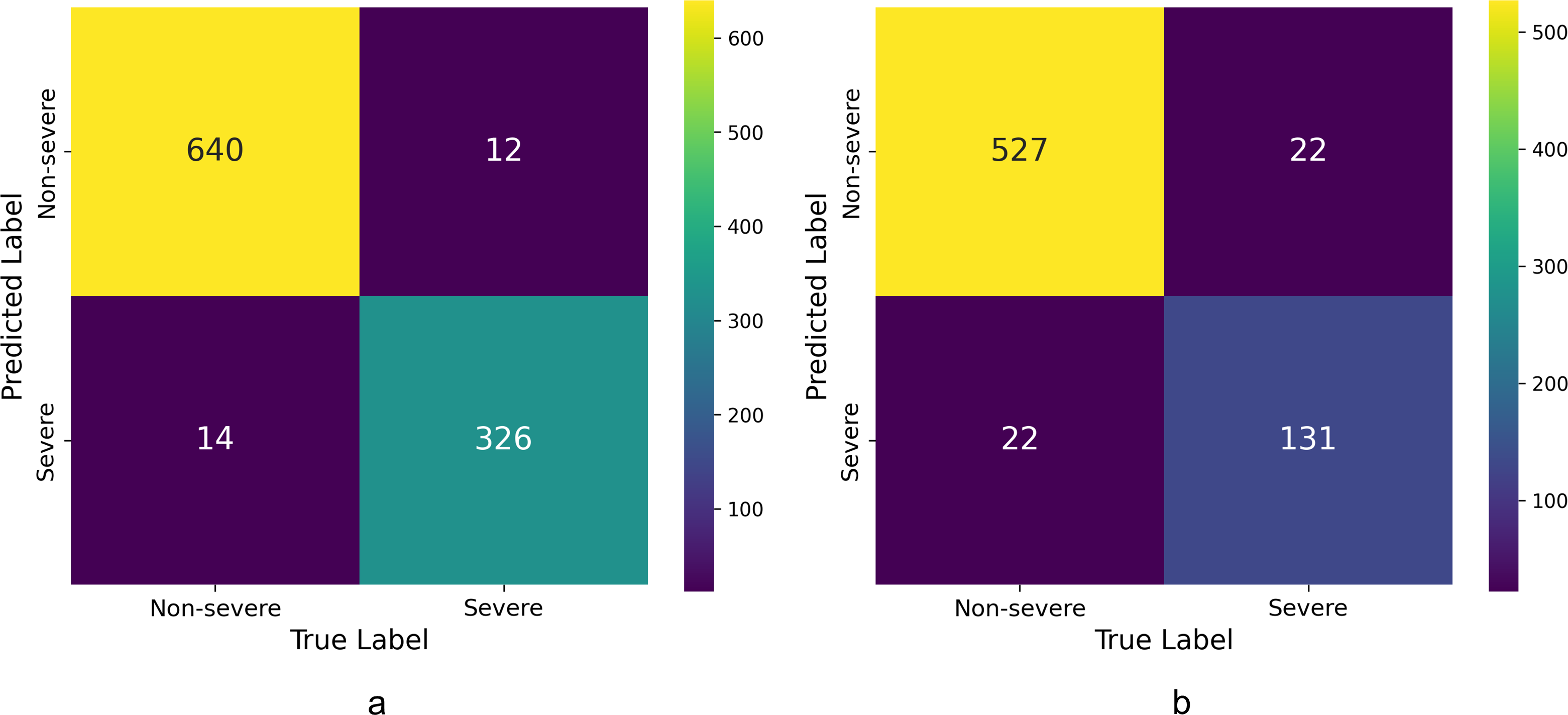

Supplement: Supplementary file 3 — Supplementary Material 3 [file 12880_2025_1641_MOESM3_ESM.tif]

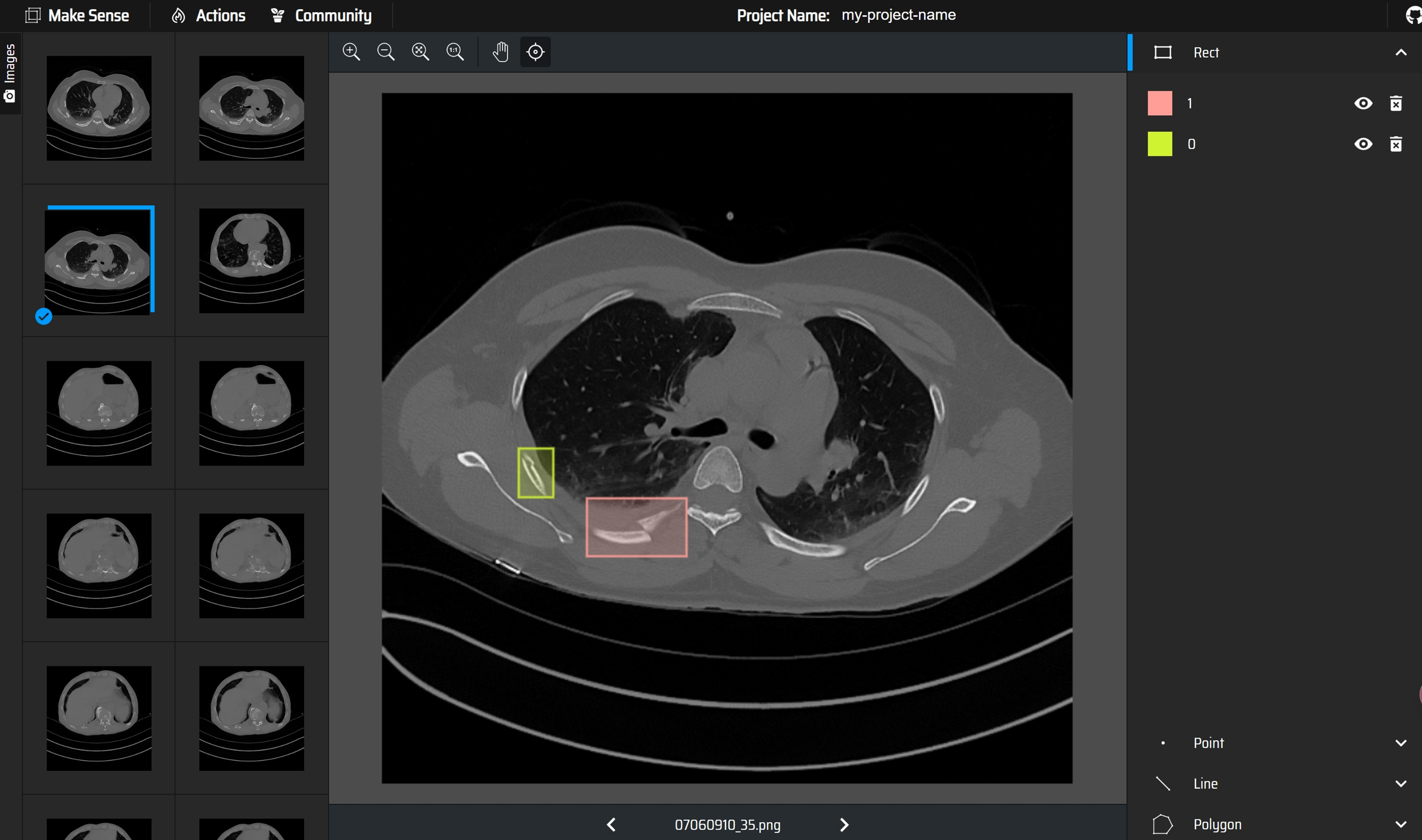

Supplement: Supplementary file 4 — Supplementary Material 4 [file 12880_2025_1641_MOESM4_ESM.tif]

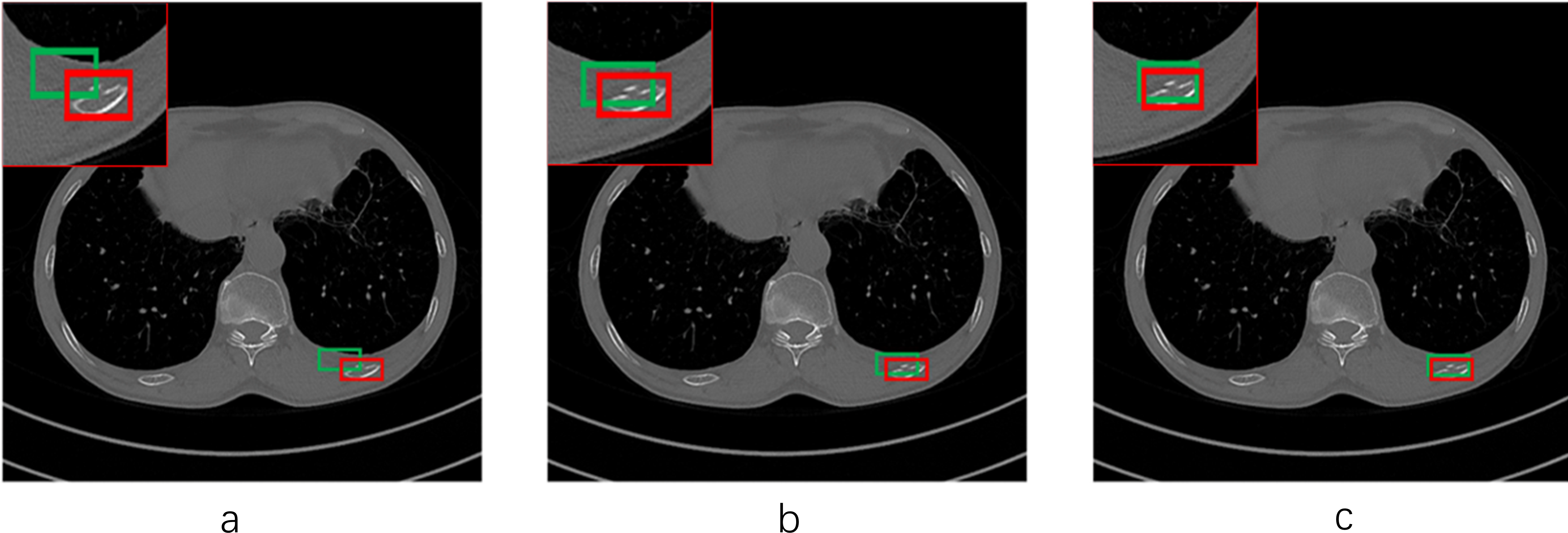

Supplement: Supplementary file 5 — Supplementary Material 5 [file 12880_2025_1641_MOESM5_ESM.tif]
